# Lentivirus-Mediated ERK2 siRNA Reduces Joint Capsule Fibrosis in a Rat Model of Post-Traumatic Joint Contracture

**DOI:** 10.3390/ijms141020833

**Published:** 2013-10-17

**Authors:** Fengfeng Li, Shen Liu, Cunyi Fan

**Affiliations:** Department of Orthopaedics, Sixth Affiliated People’s Hospital, Shanghai Jiaotong University, 600 Yishan Road, Shanghai 200233, China; E-Mails: fengmale@mail.sh.cn (F.L.); liushensjtu@sjtu.edu.cn (S.L.)

**Keywords:** post-traumatic joint contracture, extracellular signal-regulated kinase 2, ERK2, siRNA, lentivirus

## Abstract

Extracellular signal-regulated kinase (ERK)-2 is presumed to play an important role in the development of post-traumatic joint contractures. Using a rat injury model, we investigated whether treatment with ERK2 small interfering RNA (siRNA) could reduce the extent of joint capsule fibrosis after an induced injury. Rats were separated into three groups (*n* = 32 each): non-operated control group, operated contracture group and contracture-treatment group. Stable post-traumatic joint contracture was created through surgical intra-articular joint injury followed by eight weeks of immobilization. In the contracture-treatment group, the rats were treated with lentivirus (LV)-mediated ERK2 siRNA at days 3 and 7 post-surgery. The posterior joint capsule was assessed by western blotting, immunohistochemistry and biochemical analysis for changes in ERK2, phosphorylated (p)-ERK2, myofibroblast, total collagen and relative collagen Type III expression level. Biomechanical testing was used to assess the development of flexion contractures. Statistical analysis was performed using an analysis of variance. In the operated contracture group, rats that developed flexion contractures also showed elevated phosphorylated p-ERK2 expression. In the contracture-treatment group, ERK2 siRNA significantly reduced p-ERK2 expression levels, as well as the severity of flexion contracture development (*p* < 0.01). Myofibroblast numbers and measurements of total collagen content were also significantly reduced following ERK2 siRNA (*p* < 0.01). Relative collagen type III expression as a proportion of total of Types I and III collagen, however, was significantly increased in response to ERK2 siRNA (*p* < 0.01). Our findings demonstrate a role for ERK2 in the induction of joint capsule fibrosis after injury. Furthermore, we show that development of flexion contractures and the resultant increase of joint capsule fibrosis can be reduced by LV-mediated ERK2 siRNA treatment.

## 1. Introduction

Joint contracture of the elbow is a common secondary complication caused by prolonged immobility of the joint following treatment or injury. It results in an approximate 10%–15% reduction in joint motion and therefore usually requires revision surgery [1,2]. The elbow joint capsule controls the functional range of motion of the joint and is the site that frequently develops contracture [3,4]. Some nonsurgical treatments have been examined for their efficacy in preventing joint contractures [5–8]. While these treatments have achieved a certain level of success, joint contractures generally remain an unsolved problem. Therefore, there is still a need to develop a new strategy for the treatment of joint contractures.

RNA interference (RNAi) is the process by which double-stranded RNA triggers the destruction of mRNAs sharing the same sequence. It is initiated by small interfering RNAs (siRNAs), comprising short duplexes of 19–23-nucleotide single-stranded RNAs that direct the degradation of the target RNA [9–11]. Based on their significant and long-lasting inhibitory effects, siRNAs have proven to be of great value in the treatment of many joint diseases, including osteoarthritis, rheumatoid arthritis and periprosthetic osteolysis [12–14]. Previously, we showed that the local delivery of extracellular signal-regulated kinase (ERK)2 siRNA using a lentivirus (LV) could effectively and safely ameliorate the formation of joint adhesion in a rat model [15]. This result reflected the predominant role of ERK2 in joint adhesion formation. Since joint adhesion and joint capsule fibrosis are thought to form via similar mechanisms, we hypothesized that phosphorylated ERK2 may also be increased in joint capsule fibrosis and that inhibiting the phosphorylation of ERK2 may represent a promising treatment strategy. In the present study, LV-mediated ERK2 siRNA was used to inhibit ERK2 expression and phosphorylation in a model of joint capsule fibrosis.

## 2. Results and Discussion

A total of 96 rats were used in this study and 91 rats were included in the final analysis. Five animals were excluded for the following reasons: one rat in the contracture-treatment (CNT) group experienced an intra-operative tibial fracture; two rats in the operated contracture (ORC) group were euthanized because of immobilization failure at 5 days post-operatively; one rat in the non-operated control (CON) group was euthanized for failure to thrive after surgery; and one rat in the CNT group was excluded secondary to a tibial fracture encountered during biomechanical testing. All rats in the CNT group tolerated the siRNA injections. None of the rats showed evidence of wound dehiscence or deep infection across the three groups.

**Figure 1:**
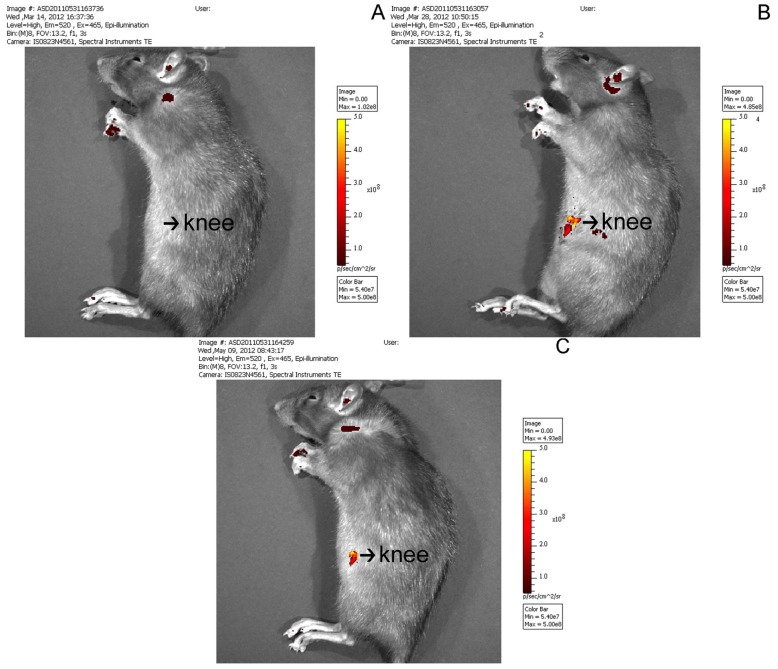
(**A**) Fluorescence images of the operated contracture (ORC) group; (**B**,**C**) Representative fluorescence images of rat tissue in the contracture-treatment (CNT) group at 2 weeks after operation (**B**) and 8 weeks after operation (**C**).

**Figure 2:**
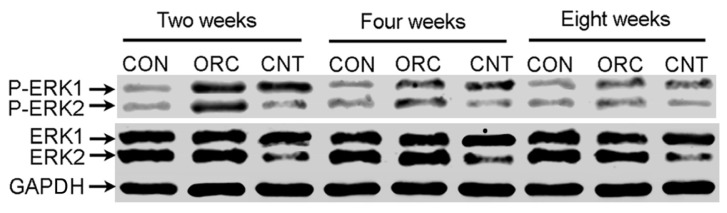
The relative ERK1/2 and phosphorylated p-ERK1/2 protein levels of the posterior joint capsule in the three groups at 2, 4 and 8 weeks after operation as determined by western blotting.

No fluorescence was detected in the joints of rats in the ORC group (Figure 1A), as expected. However at both 2 and 8 weeks after surgery, luciferase fluorescence was detected in the joints of rats in the CNT group (Figure 1B,C, respectively). This result demonstrated the successful local transfer of ERK2 siRNA. Western blot analysis further confirmed that this local delivery of LV-mediated ERK2 siRNA specifically inhibited ERK2 protein expression levels, without any marked change in glyceraldehyde-3-phosphate dehydrogenase (GAPDH) expression. At 2 weeks, the posterior joint capsule from rats in the ORC group showed a significant increase in p-ERK2 expression as compared with that in the CON group. This expression decreased by 4 weeks, and further still by 8 weeks (Figure 2).

**Figure 3:**
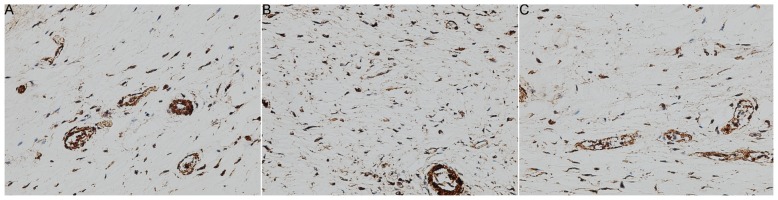
Representative immunohistochemical images (200×) of the posterior joint capsule in (**A**) the non-operated control (CON) group; (**B**) the ORC group and (**C**) the CNT group. Cell nuclei associated with α-SMA were counted as myofibroblasts.

Figure 3 and Table 1 summarize the immunohistochemistry data for all three groups. The mean myofibroblast count in the CON group was 81 ± 15 cells per high-power field. Following joint contracture surgery, the mean myofibroblast count significantly increased to 253 ± 47 cells in the ORC group, which represents a 212.3% increase in cell number (*p* < 0.01). With ERK2 siRNA treatment in the CNT group, however, the mean myofibroblast numbers were not significantly different to that in the CON group (*p* > 0.05), with 63 ± 14 cells measured per high-power field (*p* < 0.01 as compared with the ORC group). This result illustrates that ERK2 siRNA can reduce the production of myofibroblasts in the joint capsule following surgically induced joint contracture.

The flexion contracture angles for the three groups are shown in Table 2. For rats in the CON group, the mean baseline flexion contracture was 28.1° ± 3.3°. For rats in the ORC group, the mean flexion contracture increased to 124.0° ± 12.3° (341.3% increase; *p* < 0.01). However, this was significantly reduced to 41.2° ± 5.6° in the CNT group following treatment with ERK2 siRNA (*p* < 0.01, compared with the ORC group; *p* > 0.05, compared with the CON group). The weights of the rats did not significantly influence the biomechanical contracture values.

**Figure 4:**
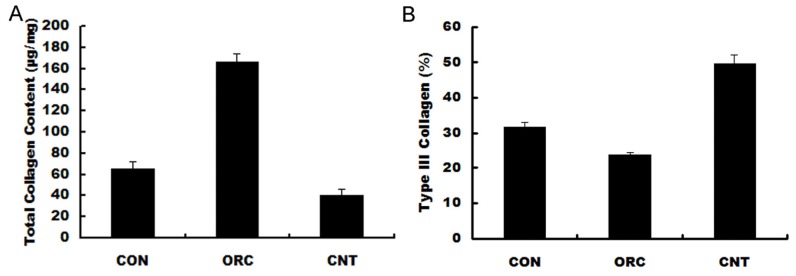
Results of biochemical analyses. (**A**) Total collagen content (μg/mg dry weight); (**B**) Type III collagen (COL3) expression relative to total of Types I and III collagen levels.

As shown in Figure 4, total collagen content in the posterior joint capsule was significantly increased in the ORC group (161.8 ± 7.9 μg/mg) compared with that in the CON group (67.3 ± 5.2 μg/mg; *p* < 0.01). This total collagen content following surgery was significantly reduced by ERK siRNA treatment in the CNT group (34.7 ± 8.6 μg/mg; *p* < 0.01). The relative expression of Type III collagen, as a proportion of total of Types I and III collagen expression, was significantly reduced in the ORC group (25.6% ± 0.7%) compared with that in the CON group (33.8% ± 1.4%; *p* < 0.01). However, this decrease observed in the ORC group was reversed following ERK2 siRNA treatment in the CNT group (46.4% ± 2.0%, *p* < 0.01 compared with the ORC group).

The development of permanent joint contracture is a complex process believed to be caused by the excessive deposition of extracellular matrix components, such as collagen, and an increase in myofibroblast hyperplasia. These tissue changes are two of the main characteristics of tissue fibrosis, and can be found in numerous conditions, such as Dupuytren’s contracture of the hand, adhesive shoulder capsulitis, scleroderma and hypertrophic wound healing [16–18].

Transforming growth factor-beta 1 (TGF-β1), which plays a very important role in collagen expression [19], has also been widely implicated in the development of tissue fibrosis. Certainly, in both human and animal models of post-traumatic joint contracture, TGF-β1 expression is increased in the joint capsule [20]. In addition, TGF-β1 has a strong mitogenic effect on NIH3T3 fibroblasts, leading to cell proliferation and the formation of fibrosis through the activation of ERK1/2 [21]. We previously demonstrated that ERK2 plays crucial roles in suppressing collagen expression and cell proliferation in TGF-β1-activated fibroblasts harvested from joint adhesion tissue in rats [22]. Furthermore, we recently demonstrated that the local delivery of a LV-mediated ERK2 siRNA ameliorated joint adhesion formation effectively and safely in a rat model [15]. Thus, we speculated that ERK2 may also play a predominant role in the onset of joint capsule fibrosis and the development of joint contracture. In this study, we identified an increase in ERK2 phosphorylation levels in rats with surgically induced joint capsule fibrosis, and further showed that inhibition of this ERK2 phosphorylation with lentivirus-mediated ERK2 siRNA could reduce the severity of joint contracture in these rats. These positive effects of ERK2 siRNA were confirmed *in vivo* through measurements of joint contracture angle and collagen content, as well as via immunohistochemistry.

The lentivirus gene delivery system offers several advantages over other viral or non-viral gene delivery systems, such as high infection efficiency; a wide variety of target cells (including dividing and non-dividing cells); a long-term infection, owing to gene integration into the chromosome of host cells; and the absence of toxicity or immune responses [23–26]. In this study, our lentivirus system efficiently delivered ERK2 siRNA into the periarticular tissue of rats, with robust effects observed, even after 8 weeks.

Patients with chronic post-traumatic elbow contracture show a marked thickening of the joint capsule and a reduced range of motion as compared with their unaffected elbows [27,28]. Indeed, histological preparations have demonstrated significant myofibroblast hyperplasia in contracted capsular tissue [20]. In addition, studies show that elbow motion in the flexion-extension arc is inversely proportional to the number of myofibroblasts in the joint capsule [29]. The myofibroblast is a specialized contractile cell of the fibroblast lineage, characterized by the expression of alpha-smooth muscle actin (α-SMA) [30]. Collagen Types I and III are also major synthetic products of myofibroblasts [31]. The relative proportions of collagens Type I and III as a percentage of the total collagen are key factors in determining the mechanical strength of repaired tissues, with a higher proportion of Type III collagen assumed to decrease the strength of the tissue by reducing fibril diameter; others report that this closely correlates with connective tissue strength [32–34]. Thus, an analysis of the histological and biochemical alterations in the surrounding tissues could serve as an indicator of joint contracture outcomes in patients.

Recently, a rabbit model was designed to study the post-traumatic contracture formation. In this model, the combination of intra-articular injury and 8 weeks of immobilization led to the development of permanent knee flexion contracture, in spite of prolonged periods of remobilization [35]. The rabbits treated with this model also showed excessive myofibroblast hyperplasia and collagen deposition in the joint capsule [36]. In our study, we mimicked this rabbit model using rats. These changes to the joint capsule parallel those changes found in humans and support the use of this surgical induction of joint fibrosis to further study the formation of post-traumatic joint contracture.

## 3. Experiment Section

### 3.1. Lentiviral Vector Construction, Virus Production and Transfection

The pshRNA-H1-Luc lentivector (System Biosciences, Mountain View, CA, USA) used in this study was designed to coexpress luciferase cloned from the copepod. The siRNA sequence targeting rat ERK2, 5′-GCACCTCAGCAATGATCAT-3′, has been shown previously to efficiently down-regulate rat ERK2 expression [22]. Pairs of complementary oligonucleotides containing these sequences were synthesized (Invitrogen, Carlsbad, CA, USA) and cloned into the pshRNA-H1-Luc lentivector. The pshRNA-H1-Luc lentivectors containing the shRNA sequences using Lipofectamine™ 2000 (Invitrogen, Beijing, China) and pPACK Packaging Plasmid Mix (System Biosciences, Shanghai, China) were cotransfected into 293T producer cells. Viral supernatants were harvested after 48 h, and the titers were determined with serial dilutions of concentrated lentivirus.

### 3.2. Group Allocation

Ninety-six female Lewis rats with weight from 220 to 280 g, aged 12 weeks, were purchased from the Shanghai Laboratory Animal Center (Chinese Academy of Sciences). All experimental procedures were approved by the authors’ Institutional Animal Review Committee. The rats were randomly assigned to one of three groups (*n* = 32 each): the ORC group; CNT group, and CON group. In the ORC and CNT groups, stable post-traumatic joint contracture was created through surgical intra-articular joint injury followed by 8 weeks of immobilization, as previously described [37,38]. Rats in the CNT group were then administered with an intra-articular injection of 0.1 mL culture medium containing the LV-mediated ERK2 siRNA at days 3 and 7 post-surgery. Rats in the CON group received no surgical or pharmacological intervention.

### 3.3. Joint Interventions

**Figure 5:**
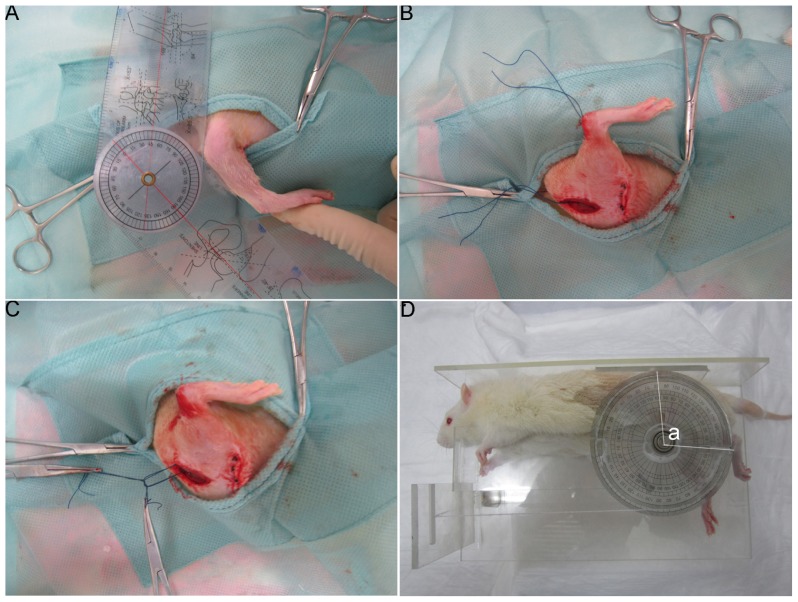
Rat model of joint contracture and measurement of the flexion contracture angle. (**A**) The knee was hyperextended to −45° to disrupt the posterior capsule; (**B**,**C**) The knee joint was immobilized at 140° flexion with Ethibond Excel polybutylate-coated braided polyester sutures; (**D**) The flexion contracture angle was measured and determined as “a”.

Under inhalation anesthesia (2%–3% isoflurane and oxygen), rats were placed in the supine position and prepared for surgery under aseptic conditions. A midline skin incision was made in the right knee joint and a lateral parapatellar arthrotomy was performed. The patella was reflected medially and the knee joint flexed to expose the femoral condyles. Two 1.5 mm × 1.5 mm cortical windows were removed from the non-articulating cartilaginous regions of the medial and lateral femoral condyles using a 1.5 mm drill bit. The anterior cruciate ligament and posterior cruciate ligament were then sequentially incised and the knee hyperextended to −45° to disrupt the posterior capsule (Figure 5A). The right knee was immobilized at 140° of flexion with Ethibond Excel polybutylate-coated braided polyester sutures (Figure 5B,C). The proper reduction of the patellofemoral joint was checked prior to closure. After surgery, rats were permitted unrestricted movement within their cages (10 rats to a cage of 0.1 m^3^).

### 3.4. *In Vivo* Bioluminescence Assay

Bioluminescence assays comprise a high-sensitivity and non-invasive technique for monitoring specific cellular and genetic activities in a living organism. At 2 and 8 weeks after surgery, the luciferase expression and distribution in the individual rats in the CNT group were measured using a Xenogen IVIS 50 Bioluminescence System (R&D Systems). For a comparison, fluorescence was also detected in the joint of rats in the ORC group.

### 3.5. Western Blot

At 2, 4 and 8 weeks after the initial surgery date, a total of 45 rats weresacrificed using an overdose of pentobarbital sodium (Dainihon, Osaka, Japan). Posterior joint capsules were harvested and lysed with lysis buffer. The cell lysates were centrifuged at 13,000× *g* for 15 min at 4 °C, and the supernatants were collected for western blot analysis. Equal amounts of protein were separated on a 12% gel using SDS–PAGE and electrotransferred to nitrocellulose membranes (Millipore Corp., Billerica, MA, USA). After blocking with 5% non-fat milk, the membranes were incubated with antibodies against ERK2 and p-ERK (Cell Signaling Technology, Beverly, MA, USA) for 1 h at room temperature. A monoclonal anti-GAPDH antibody (Cell Signaling) was used as a loading control. After washing, the membranes were incubated with HRP-conjugated IgG (Acris Antibodies, GmbH, Hiddenhausen, Germany) for 1 h, and immunoreactive bands were detected by chemiluminescence (Amersham Biosciences, Freiburg, Germany).

### 3.6. Immunohistochemistry

Single-labeling immunohistochemistry was used to identify and quantify myofibroblasts in joint capsule preparations of 8 weeks after operation, as previously described [37]. Briefly, 8 mm thick sections of frozen samples from 23 sacrificed rats at 8 weeks were cut and mounted onto pre-coated glass slides, and then pretreated with hyaluronidase. Normal goat serum (10%) diluted in PBS was applied as a blocking agent. A monoclonal α-SMA antibody (clone asm-1, Roche Molecular Biochemicals, Laval, QC, Canada) was incubated for 1 h at 37 °C. The sections were washed and then incubated for 1 h at room temperature with a sheep anti-mouse IgG horseradish peroxidase (HRP)-conjugated secondary antibody (Roche, Shanghai, China) (dilution 1:50). The sections were washed again and then incubated with DAB/peroxide substrate (Roche, Shanghai, China). Finally, the sections were counterstained with 40-6-diamidine-2-phenyl indole (DAPI; Vector Laboratories, Burlingame, MA, USA) to label all nuclei. The sections were viewed under a light microscope (Zeiss, Axioskop 2 plus, Toronto, ON, Canada) and images were captured (200×) with a digital camera (Zeiss, Axiocam, Toronto, ON, Canada). Five randomly selected areas from four sections of each specimen were used to analyze cell numbers using Image-Pro Plus (Media Cybernetics, Silver Spring, MD, USA). Cell nuclei associated with α-SMA were counted as myofibroblasts. The data were collected as the average number of myofibroblasts per high-power field.

### 3.7. Measurement of the Flexion Contracture Angle

Twenty-three Rats remaining in each group were euthanized at 8 weeks and the sutures were carefully removed. Each rat was then positioned on a table on its right side, with the left lower leg attached to a 5 cm diameter pulley with a silk thread. A thick silk thread was attached to the periphery of the pulley and an extension of 0.49 N was applied by pulling the thick silk thread with a weight. Flexion contracture angles were determined as “a” (Figure 5D). All measurements were made within 15 min after euthanasia.

### 3.8. Biochemical Analysis

Following biomechanical evaluation, the same rats were used to measure collagen content in the posterior knee joint capsule. The posterior knee joint capsule of each rat was harvested, lyophilized, and stored at −20 °C until analysis. Total collagen content was calculated as a measure of hydroxyproline content using the Woessner method [39,40]. The relative amounts of Type I and III collagens were determined using electrophoresis of alpha chains and cyanogen bromide-cleaved peptides, according to the method of Chan and Cole [41]. This ratio was expressed as a percentage of Type III collagen to total of Types I and III collagen.

### 3.9. Statistics

Statistical analyses were performed using a one-way analysis of variance (ANOVA) with a Student–Newman–Keuls *post hoc t*-test. The data are presented as the mean ± standard deviation (SD). Significance was set at *p* < 0.05. All statistical analyses were conducted using SPSS 11.0 (SPSS Inc., Chicago, IL, USA).

## 4. Conclusions

The results of this study suggest that ERK2 activation is an important event in the pathogenesis of joint capsule fibrosis and the subsequent loss of motion after intra-articular injury. This study provides a novel and promising strategy to prevent the development of joint capsule fibrosis, although further studies involving larger animals are required to support these results.

## Figures and Tables

**Table 1:** Myofibroblast counts in the CON, ORC and CNT groups.

Group	Number	Myofibroblasts (hpf)	a*p*	b*p*
CON	8	81 ± 15	NA	<0.01
ORC	8	253 ± 47	<0.01	NA
CNT	7	63 ± 14	>0.05	<0.01

A total of 23 animals in three groups were used for this evaluation; Data represent means ± SD; Values of *p* < 0.05 are considered to indicate statistical significance;

a *p*, *vs*. CON;

b *p*, *vs*. ORC;

NA, not applicable.

**Table 2:** Contracture angles of the rats in the CON, ORC and CNT groups.

Group	Number	Contracture angles (°)	a*p*	b*p*
CON	8	28.1 ± 3.3	NA	<0.01
ORC	7	124.0 ± 12.3	<0.01	NA
CNT	8	41.2 ± 5.6	>0.05	<0.01

A total of 23 animals in three groups were used for this evaluation; Data represent means ± SD; Values of *p* < 0.05 are considered to indicate statistical significance;

a *p*, *vs*. CON;

b *p*, *vs*. ORC;

NA, not applicable.
